# Optimization Design of the Two-Stage Reduction Micro-Drive Mechanism Based on Particle Swarm Algorithm †

**DOI:** 10.3390/mi16070826

**Published:** 2025-07-19

**Authors:** Na Zhang, Dongmei Wang, Kai Li, Kaiyang Wei, Hongyu Ge, Manzhi Yang

**Affiliations:** 1 The Art College, Xi’an University of Science and Technology, Xi’an 710054, China; nazhang@xust.edu.cn (N.Z.); wangdongmei@163.com (D.W.); 2 College of Mechanical Engineering, Xi’an University of Science and Technology, Xi’an 710054, China; lk149941@163.com (K.L.); ky15293595762@163.com (K.W.); ghy_xkd@sohu.com (H.G.)

**Keywords:** two-stage reduction, micro-drive mechanism, particle swarm algorithm, optimization design

## Abstract

Achieving high-precision positioning operations in a small space was of great significance in aerospace, biomedical, and other fields. In order to obtain smaller displacements with higher accuracy, this paper focused on the design, optimization, and performance analysis of a two-stage reduction micro-drive mechanism. Using the principle of lever and the principle of balanced additional force, a two-stage reduction micro-motion mechanism without parasitic motion and non-motion directional force was designed, and the structure optimization of the mechanism was completed by employing the particle swarm algorithm. A finite element analysis was conducted to assess the strength, dynamics, and kinematic properties of the mechanism. Experimental methods were also employed to analyze its dynamic and kinematic properties. The analysis results demonstrated that the mechanism met the design requirements in terms of strength and dynamic properties, with a maximum error of 9.02% and a maximum kinematic error of 0.0267 μm. The achieved reduction ratio was 24.73:1. These results indicated that the mechanism possesses excellent strength and dynamic performance, a large reduction ratio, high motion accuracy, and good linearity. This paper contributes significantly to the advancement of research in precision mechanical motion and micro-drive mechanisms.

## 1. Introduction

With the increasing demand for precision mechanical positioning systems in high-precision machine tools [1], precision optics [2], additive manufacturing [3,4], and other high-tech fields, the manufacturing of mechanical equipment has imposed higher requirements on the motion accuracy [5] and machining precision of mechanical systems [6,7]. Precision positioning operations that achieve high precision in a small space have become necessary research in the field of high-precision technology [8]. The micro-drive mechanism [9] could be used independently to obtain precise output [10]. It could also be combined with a macro-drive to form a macro–micro dual-drive system [11,12], enabling macro–micro motion with a large stroke [13,14] and high precision [15]. The micro-drive mechanism realized various functions, such as linear transmission [16,17,18], displacement amplification [19], displacement reduction [20] (accuracy improvement), and displacement conversion [21] (converting high-precision straight-line motion into high-precision rotation). As a result, micro-drive plays an important role in aerospace, defense, optics, machinery, electronics, and other fields [22,23].

The micro-drive reduction mechanism plays a crucial role in improving the accuracy and expanding the application field of micro-drive systems, enabling high-precision micro-drive operations in small spaces. Thus, the research on micro-drive reduction mechanisms holds significant importance [24,25,26,27,28]. Rui Lin et al. [29] proposed a flexible decoupled XY high-precision large-range positioning platform. Through a finite element analysis, the platform’s positioning range, resonant frequency, and safety were evaluated. The experimental results showed that the platform achieved millimeter-level positioning and was capable of high-speed positioning over a large range. Sakuma S et al. [30] designed a mechanism by combining springs with different stiffness levels driven by magnetic force; the prototype device’s performance was evaluated, and the results indicated a standard deviation of probe tip displacement below 0.18 μm for repeatable positioning accuracy. These findings highlight that the first-level reduction micro-drive mechanism could achieve micro-operations within a small spatial range. However, its motion accuracy was insufficient for more precise micro-operations, necessitating higher reduction ratios and motion accuracy. Therefore, studying the second-level reduction fretting mechanism becomes particularly important.

Optimizing the micro-mechanism facilitates the attainment of an optimal target structure [31,32]. Chen FX et al. [33] designed a piezoelectric-driven dual-drive linear motion system and used a genetic algorithm to optimize the mechanism’s structure. Their experimental results showed an optimized amplification ratio of 91 and a travel distance of 6.13 mm. Huang SC et al. [34] designed an XY positioning platform based on flexure hinges, utilizing composite leaf springs and straight circular flexure hinges. The mechanical optimization of the positioning platform was performed using the response surface method of finite element analysis, resulting in improved static and dynamic characteristics. Based on the above analysis, it is evident that in order to achieve the minimum reduction ratio and greater motion accuracy, the two-stage reduction mechanism could be optimized by minimizing the reduction ratio while maintaining a constant structural space. The two-stage reduction micro-drive mechanism with the minimum reduction ratio in a finite space could offer enhanced motion accuracy and a smaller motion space, providing advantages in the application field of reduction fretting mechanisms.

In conclusion, it was crucial to optimize the structure of the two-stage reduction mechanism with the minimum reduction ratio. Based on the flexible hinge principle, this paper presented a symmetrical structure of a two-stage reduction mechanism utilizing the lever principle and the principle of balanced additional force. The particle swarm algorithm was employed to optimize the mechanism’s structure under size constraints, resulting in the highest precision and the maximum reduction ratio within a given space. The piezoelectric ceramic actuator and the two-stage reduction micro-drive mechanism serve as the micro-driver and the actuator, respectively, in constructing the two-stage reduction micro-drive system. The related properties were studied, and the results demonstrated an excellent performance of the mechanism. An experimental platform was established for verification, and a comparison between the finite element analysis results and theoretical results confirmed that the design of this mechanism is excellent.

The rest of this article consists of the following: The second chapter discusses the completion of the optimization design of the two-stage reduction micro-drive mechanism. In the third chapter, the performance analysis of the mechanism is discussed. In the fourth chapter, the performed experiment and the mechanism are discussed. In the fifth chapter, the full text is summarized.

## 2. Optimization Design 

### 2.1. Design of Mechanism

#### 2.1.1. Lever Principle

The lever principle is also known as the “lever balance condition”. The magnitude of the two forces acting on the two ends of the lever (power point and resistance point) is inversely proportional to the length of their moment arm. Power × power arm length = resistance × resistance arm length, expressed by the following algebraic formula:

(1) $$F_{1}\cdot L_{1}=F_{2}\cdot L_{2}\;$$

**Figure 1:**
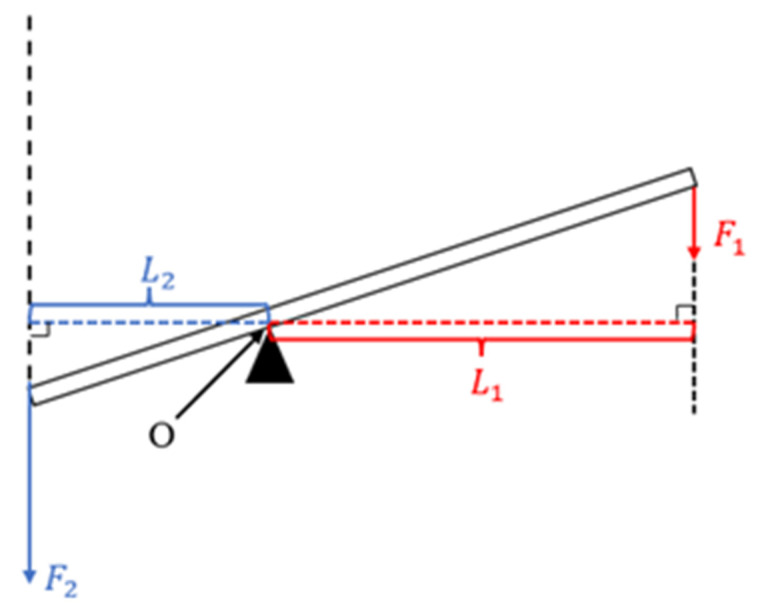
Lever schematic.

In the formula, $F_{1}$ represents the power, $F_{2}$ represents the resistance, $L_{1}$ represents the length of the power arm, and $L_{2}$ represents the length of the resistance arm. Figure 1 shows a schematic diagram of the lever principle. The detailed principle can be found in Section 2.3.1 of reference [19] (published by our research group).

If the power arm is less than the resistance arm, the power > the resistance, and this lever plays a role in reduction. The reduction mechanism designed in this article was based on the lever principle.

#### 2.1.2. Principle of Balanced Additional Force

**Figure 2:**
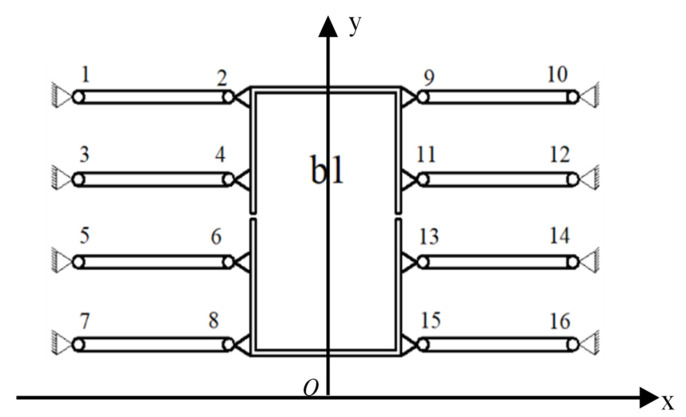
Principle of balanced additional force.

The principle of balanced additional force is shown in Figure 2. The micro-driver was fixedly connected to b1, and the mechanism was symmetrically designed with eight flexible hinge [35] components (flexible hinge 1–16). When the driver produces a drive displacement, the flexible hinge will be deformed. In addition to the force of the main direction of motion, forces in non-directions of motion are generated. At this time, the symmetrically distributed eight flexible hinge components have equal magnitude and opposite directions of force in the non-moving direction due to deformation, and the transverse forces from both directions can be balanced, respectively, to achieve the additional force balance. The principle of balanced additional force can be found in Section 2.3.2 of reference [19] (published by our research group).

#### 2.1.3. Initial Mechanism Design

The two-stage reduction micro-drive mechanism was designed based on the principle of flexible hinge using levers, the mechanism designed reduction ratio was 4:1, and the material used was 60Si2Mn spring steel. The parameters of material are shown in Table 1.

**Figure 3:**
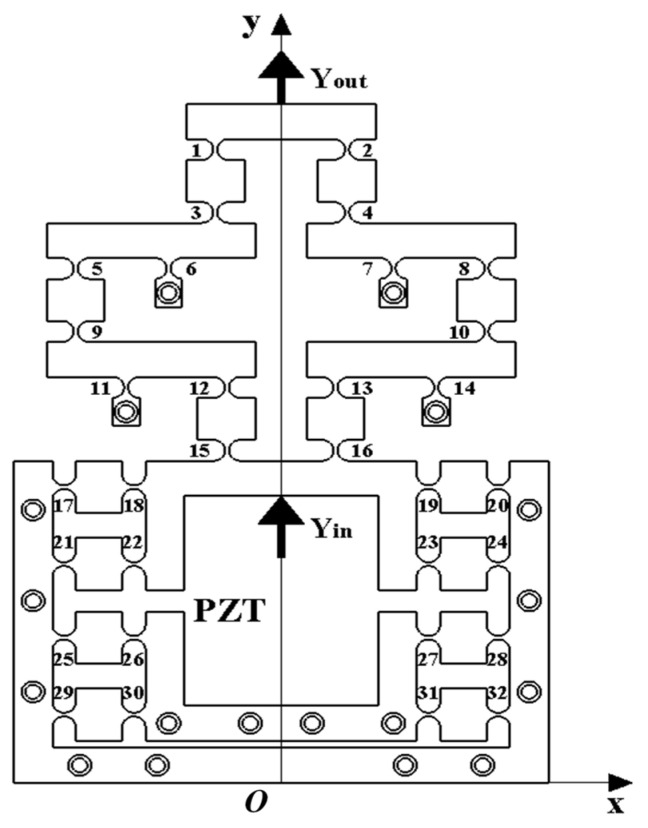
Working principle of mechanism.

The mechanism was driven by a piezoelectric ceramic actuator, and the overall size of the micro-drive mechanism was 138 mm × 194 mm × 50 mm. Eighteen M6 threaded holes and screws were used to fix the mechanism on the workbench, and 32 hinges were symmetrically arranged on it to eliminate parasitic motion. The working principle is shown in Figure 3.

The piezoelectric ceramic brake (PZT) generates input displacement Y_in_, which was transmitted to the first stage reduction structure by flexure hinge 13 and 16, and flexure hinge 14 as the fixed end. The output displacement after the first stage reduction was transmitted to the second stage reduction structure by flexible hinges 8 and 10, and flexible hinge 7 was the fixed end. The output displacement after the second stage reduction was transmitted by flexible hinges 2 and 4, and finally the reduced output displacement Y_out_ was obtained at the top of the mechanism. Among them, three holes on the right of flexure hinge 20 were used to fix the mechanism, and the holes next to flexure hinge 14 could provide a fixed effect for the first-level reduction mechanism. The hole next to flexure hinge 7 provided a holding action for the secondary reduction mechanism and the two holes on the left of flexure hinge 31 were used to fix the mechanism to ensure that the mechanism only moves forward along the Y-axis when the piezoelectric ceramic driver was driven. The bottom two holes of the mechanism were used to hold the mechanism and provide preload for the PZT during operation by thrust.

### 2.2. Reduction Ratio Calculation

Let the coordinate value of flexure hinge 13 be x_1_ (mm), the coordinate value of flexure hinge 14 be x_2_ (mm), the coordinate value of flexure hinge 10 be x_3_ (mm), the coordinate value of flexure hinge 8 be x_4_ (mm), the coordinate value of flexure hinge 7 be x_5_ (mm), and the coordinate value of flexure hinge 4 be x_6_ (mm). The initial coordinate values are shown in Table 2.

In the first-level reduction lever, flexure hinge 14 was fixed on the micro-drive mechanism using bolts, making it the fulcrum of the lever mechanism. Flexure hinges 10 and 13 serve as the two ends of the lever mechanism. According to the lever principle, the first-level reduction ratio was

(2) $$K_{1}=\frac{l_{13–14}}{l_{10–14}}=\frac{x_{2}-x_{1}}{x_{3}-x_{2}}=2$$

In the two-stage reduction lever, flexible hinge 7 was fixed on the micro-drive mechanism using bolts, making it the fulcrum of the lever mechanism. Flexible hinges 4 and 8 serve as the two ends of the lever mechanism. According to the lever principle, the two-stage reduction ratio was

(3) $$k_{2}=\frac{l_{8–7}}{l_{4–7}}=\frac{x_{4}-x_{5}}{x_{5}{-x}_{6}}=2\;$$

Therefore, the reduction ratio of the designed two-stage reduction micro-drive mechanism was

(4) $$k=k_{1}\times k_{2}=\frac{\left(x_{2}-x_{1}\right)\left(x_{4}{-x}_{5}\right)}{\left(x_{3}{-x}_{2}\right)\left(x_{5}{-x}_{6}\right)}=4\;$$

### 2.3. Structural Optimization

Due to the relatively simple nature of the problem addressed in this paper and the small computational requirements, as well as the ease of implementation and guaranteed convergence to the global optimal solution, the particle swarm optimization algorithm was selected as the optimization algorithm for the two-stage reduction micro-drive mechanism. The goal of the optimization was to maximize the reduction ratio within certain constraints of the structural space [36,37,38].

Suppose that in a D-dimensional space, an initial population of N particles was produced. Then the position vector of the ith particle could be expressed as

(5) $$x_{i}=\left(x_{i1},x_{i2},x_{i3},x_{iD}\right),i=1,2,3\cdots N$$

The velocity vector of the ith particle could be expressed as

(6) $$v_{i}=\left(v_{i1},v_{i2},v_{i3},v_{iD}\right),i=1,2,3\cdots N$$

The optimal individual extreme value currently searched by the ith particle could be expressed as

(7) $$p_{best}=\left(p_{i1},p_{i2},p_{i3},p_{iD}\right),i=1,2,3\cdots N$$

The global optimal extreme value currently searched by the whole population could be expressed as

(8) $$g_{best}=\left(g_{i1},g_{i2},g_{i3},g_{iD}\right)$$

After the two optimal extreme values were found, the particle updates its velocity and position through Equations (6) and (7), and finally obtains the optimal solution according to the requirements.

$$v_{i}\left(t+1\right)=\omega v_{i}\left(t\right)+c_{1}\times rand\left(\;\right)\times$$

$$\left({pb}_{id}\left(t\right)-x_{i}\left(t\right)\right)+c_{2}\times rand\left(\;\right)\times$$

(9) $$\left(g_{bd}\left(t\right)-x_{i}\left(t\right)\right)\times \left(g_{bd}\left(t\right)-x_{i}\left(t\right)\right)$$

(10) $$x_{i}\left(t+1\right)=x_{i}\left(t\right)+v_{i}\left(t+1\right)$$

where *t* represents the current iteration number, *ω* represents the inertia factor, *rand*() is a random number between (0, 1), c_1_ represents the individual learning factor, *c*_2_ represents the social learning factor, *pb_id_* represents the d-dimension of the local optimal solution of particle i, and *g_bd_* represents the d-dimension of the global optimal solution.

As this two-stage reduction micro-drive mechanism was symmetric on the Y-axis, the right half was selected for the optimization analysis. It can be seen from Figure 3 that the secondary reduction process was mainly completed by hinges 4, 7, 8, 10, 13, and 14, and the reduction ratio was only related to the distance between these six hinges. The mechanism only had displacement in the y-axis direction, so the optimization target was converted to find the x-axis coordinate values of these six hinges. Let the coordinate value of flexure hinge 13 be *x*_1_ (mm), the coordinate value of flexure hinge 14 be *x*_2_ (mm), the coordinate value of flexure hinge 10 be *x*_3_ (mm), the coordinate value of flexure hinge 8 be *x*_4_ (mm), the coordinate value of flexure hinge 7 be *x*_5_ (mm), and the coordinate value of flexure hinge 4 be *x*_6_ (mm).

The objective function of this problem was

(11) $$K=\frac{Y_{in}}{Y_{out}}=\frac{\left(x_{2}-x_{1}\right)\times \left(x_{4}-x_{5}\right)}{\left(x_{3}-x_{2}\right)\times \left(x_{5}-x_{6}\right)}$$

The constraint conditions were

$$5\leq x_{1}<x_{2\;}{;x}_{1}<x_{2}<x_{3}{;x}_{2}<x_{3}\leq 65;x_{4}=x_{3}$$

$$x_{6}<x_{5}<x_{4};5\leq x_{6}<x_{5}{;x}_{3}-x_{2}\geq 10;x_{5}-x_{6}\geq 10$$

In formula, *x*_1_, *x*_2_, *x*_3_, *x*_4_, *x*_5_, and *x*_6_ must be integers.

The particle swarm program was written using the Matlab 2022a software program to optimize the calculation, and the x-coordinates of the new hinges were as follows:

$$x_{1}=5,x_{2}=55,x_{3}=65$$

$$x_{4}=65,x_{5}=15{,x}_{6}=5$$

**Figure 4:**
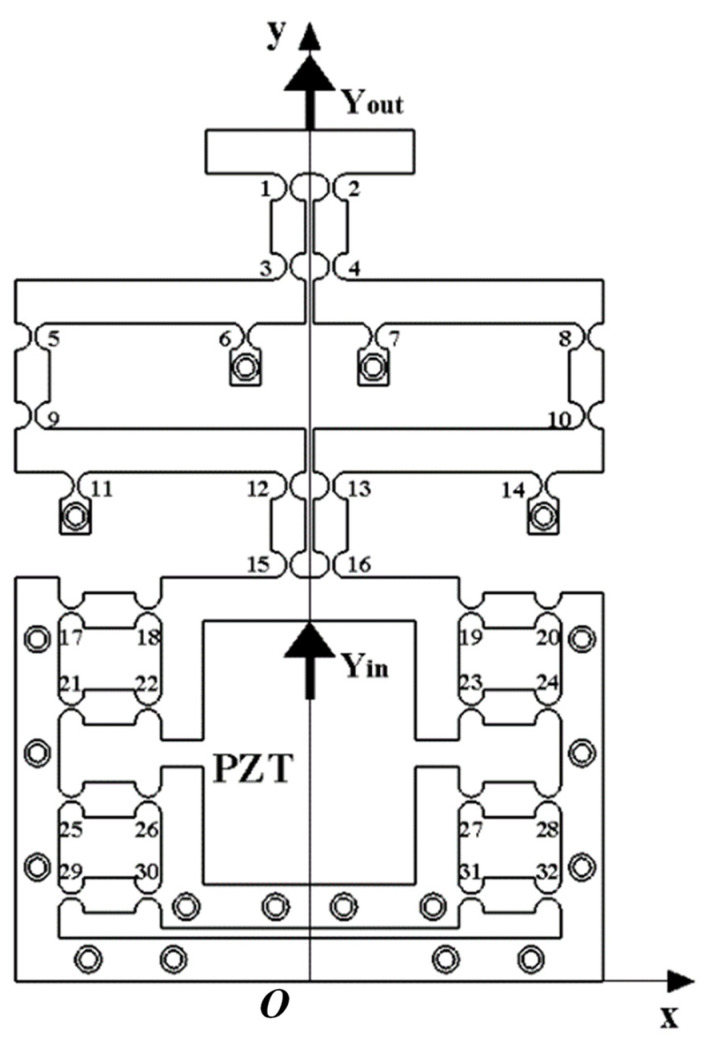
Structure diagram of optimized micro-motion mechanism.

The working principles diagram of the optimized two-stage reduced micro-drive mechanism obtained by remodeling the optimized hinge position is shown in Figure 4. Bringing the optimized value into Equation (4) yields K = 25; compared with before, the reduction ratio of two-stage reduction micro-drive mechanism has been significantly improved. Under the condition of strict constraint conditions, the optimized structure obtained a better solution than the pre-optimized one and the reduction ratio of the two-stage reduced micro-drive mechanism increased from 4 to 25.

## 3. Performance Analysis

### 3.1. Strength Analysis

The strength of the mechanism is a crucial factor that affects its safety. In this paper, the finite element method was utilized to analyze the strength of the micro-drive mechanism.

**Figure 5:**
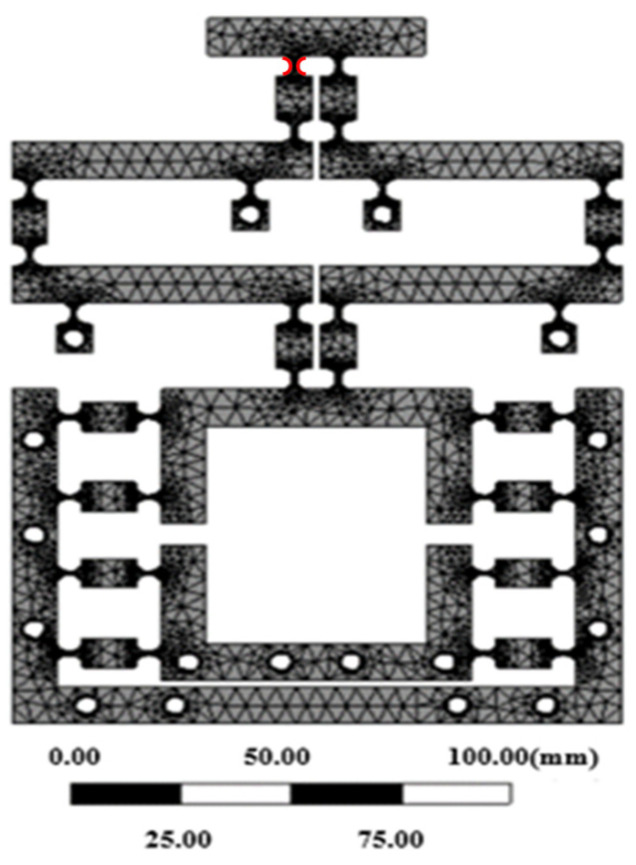
Diagram of mechanism’s meshing.

For the material properties, the 60Si2Mn parameter was selected for the finite element statics analysis. The mesh partitioning process involved initially segmenting the entire model into fully free meshes. Subsequently, 64 semicircular arcs representing the 32 flexible hinges of the mechanism were refined as mesh units using the ‘Refinement 1’ parameter. Figure 5 illustrates the grid diagram of the divided mechanism. The model was divided into 745,626 units and contained 1,126,720 nodes. The figure shows that the meshing of critical components was relatively fine and smooth, without any crossing or broken meshes, indicating good mesh quality.

**Figure 6:**
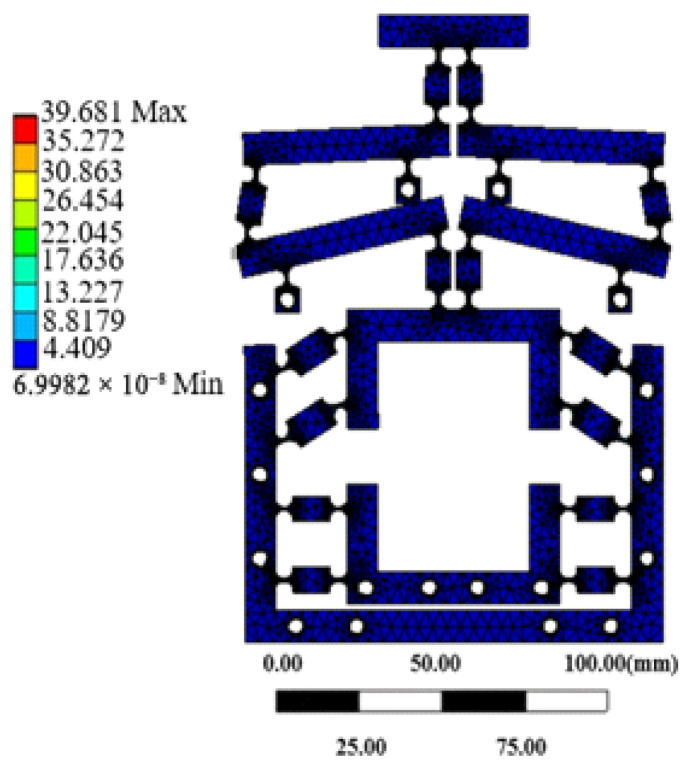
Stress nephogram of micro-drive mechanism.

The strength analysis of the micro-drive mechanism primarily focused on determining whether the mechanism would sustain damage under the influence of the micro-driver. Therefore, it was essential to analyze whether the micro-drive mechanism would withstand the maximum driving displacement of the piezoelectric ceramic actuator, which also represented the maximum simulated stress for assessing the motion of the micro-drive mechanism. The optimized micro-drive mechanism was introduced into the ANSYS-workbench 12.0 Static Module, and then fixed constraints were applied to the 18 bolt holes on the mechanism, and a positive Y-axis displacement of 15 μm was imposed at the drive position within the mechanism. The stress nephogram illustrating the results is presented in Figure 6. The maximum simulated stress experienced by the mechanism was 39.681 Mpa.

The permissible stress of the material was

(12) $$\left[\sigma \right]=\frac{\sigma_{s}}{\lambda }$$

The yield limit (*σ_s_*) of the material (60Si2Mn) was 1176 MPa. Let the safety coefficient *λ* be 1.5, and bring it into Formula (12) to find that the allowable stress [*σ*] of the material was 784 MPa. The maximum simulated stress of the mechanism was 39.681 MPa, which was less than the allowable stress of the material. The finite element analysis of statics showed that the mechanism was safe and reliable in the driving process of piezoelectric ceramic actuator, and its maximum stress met the requirements of material checking strength. Therefore, the strength of the mechanism met the design requirements.

### 3.2. Dynamic Performance

The natural frequency is a critical parameter for evaluating the dynamic performance of a micro-drive mechanism, and it helps determine whether resonance will occur during its operation. In this study, a mode analysis of the two-stage reduced micro-drive mechanism was conducted using a finite element simulation software program. The dynamics module of the finite element was utilized for free mode analysis, yielding the natural frequency results for the first six modes, as presented in Table 3.

The two-stage reduction micro-drive mechanism was driven by a P235.1s piezoelectric ceramic driver operating at a frequency of 300 Hz. From Table 3, it can be observed that the two-stage reduction micro-drive mechanism did not exhibit resonance with the piezoelectric ceramic driver during its operation. Therefore, the two-stage reduction micro-drive mechanism satisfies the design and operational requirements.

### 3.3. Motion Performance

**Figure 7:**
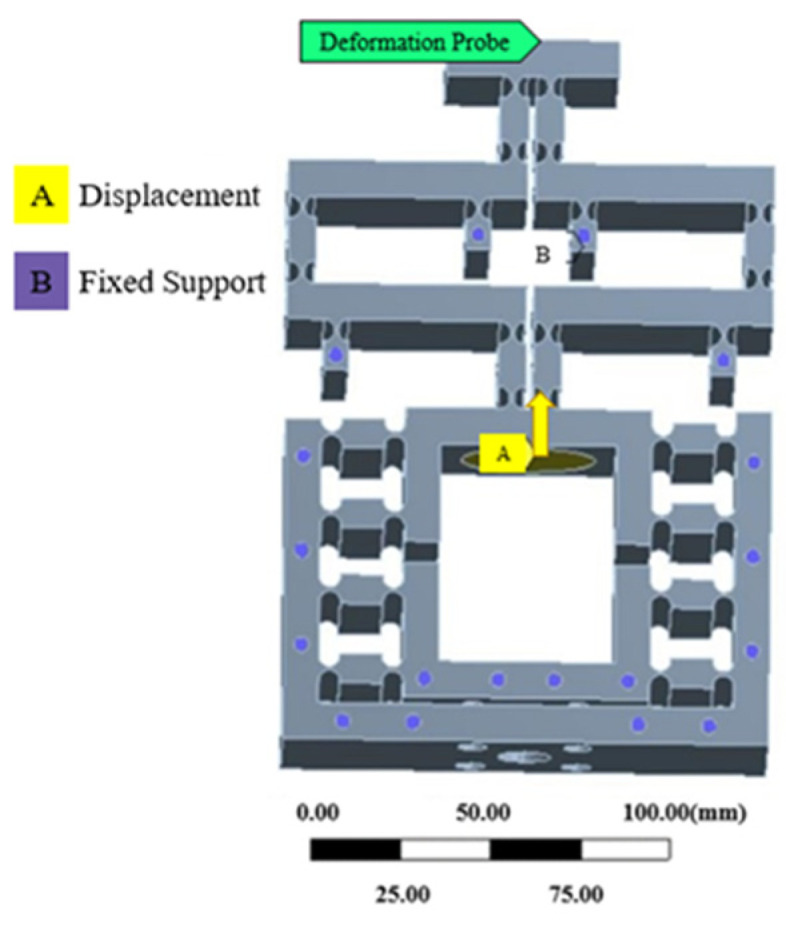
Fixed constraint and applied displacement.

**Figure 8:**
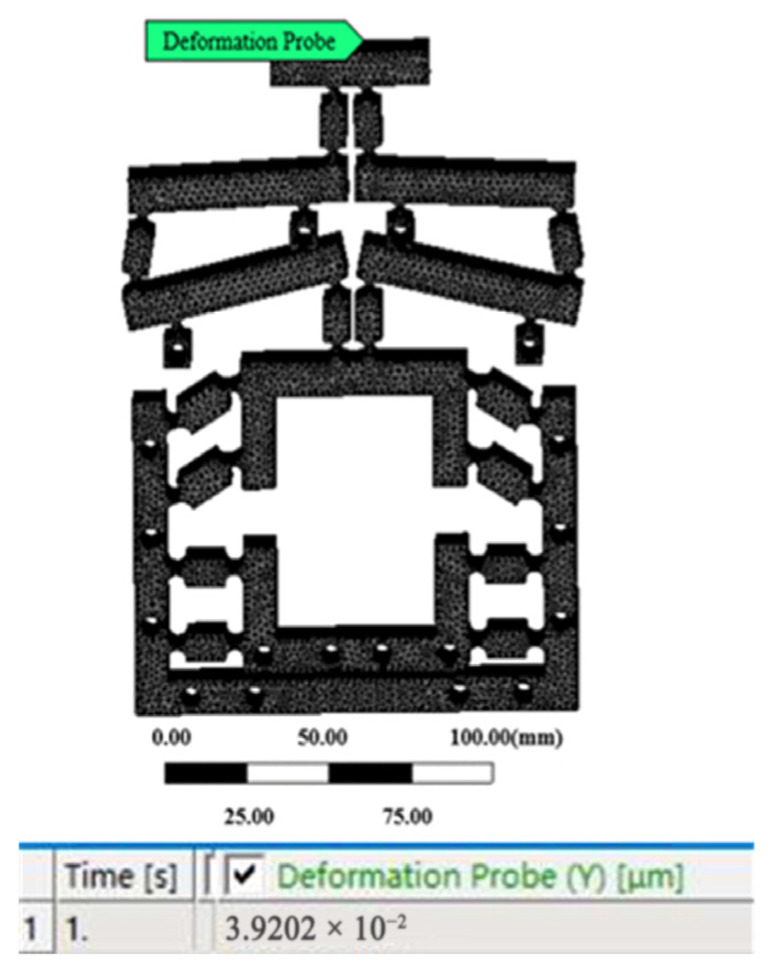
Result of 1 μm input.

The finite element meshing was consistent with Section 3.1. Applying constraints and the displacement of the output midpoint was measured by the probe function is shown in Figure 7. Taking an input displacement of 1 μm as an example, the optimized output displacement was 0.039202 μm, as shown in Figure 8. The input displacement of the mechanism was 0 μm–15 μm as the initial conditions, and the output displacement value was calculated.

## 4. Experiment and Discussion

### 4.1. Dynamic Performance Test

**Figure 9:**
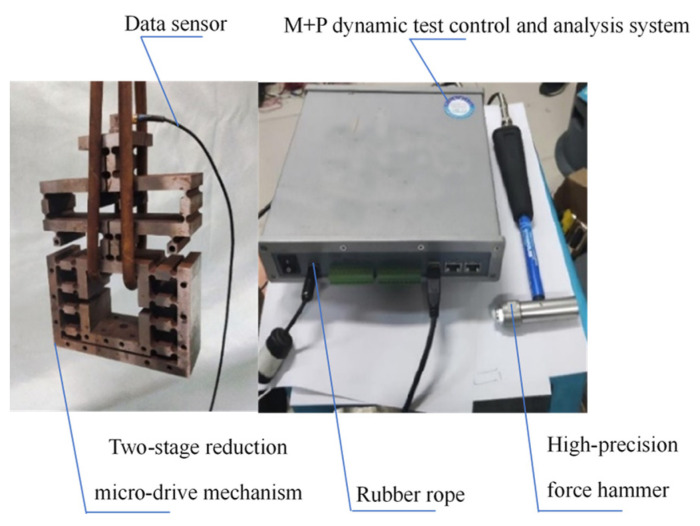
Dynamic performance test of two-stage reduction micro-drive mechanism.

**Figure 10:**
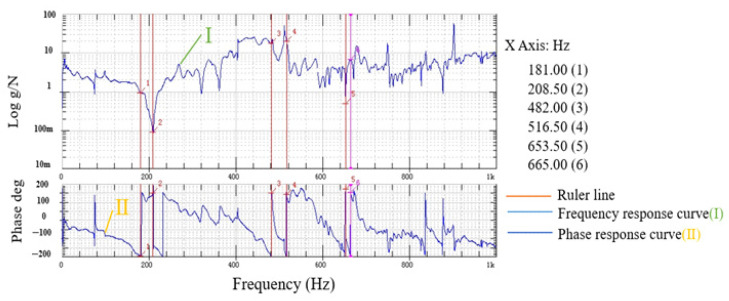
Experimental results of dynamic performance.

The natural frequency experiment of the two-stage reduction micro-drive mechanism was conducted using a setup consisting of the two-stage reduction micro-drive mechanism, a data sensor, a rubber rope, a high-precision force hammer, and the M + P dynamic test control and analysis system. The rubber rope was used to hang the mechanism freely with the data acquisition front end fixed to the mechanism. The other end of the data acquisition line was connected to the M + P dynamic test control and analysis system. The high-precision force hammer was employed to strike the mechanism, and the resulting experimental data was displayed on the computer. The experimental setup and dynamic performance of the two-stage reduction micro-drive mechanism were depicted in Figure 9, while the experimental results of the dynamic performance were presented in Figure 10. The first six natural frequencies of the two-stage reduction micro-drive mechanism were analyzed using both finite element simulations and experimental measurements, and the results are summarized in Table 4.

### 4.2. Motion Performance Test

**Figure 11:**
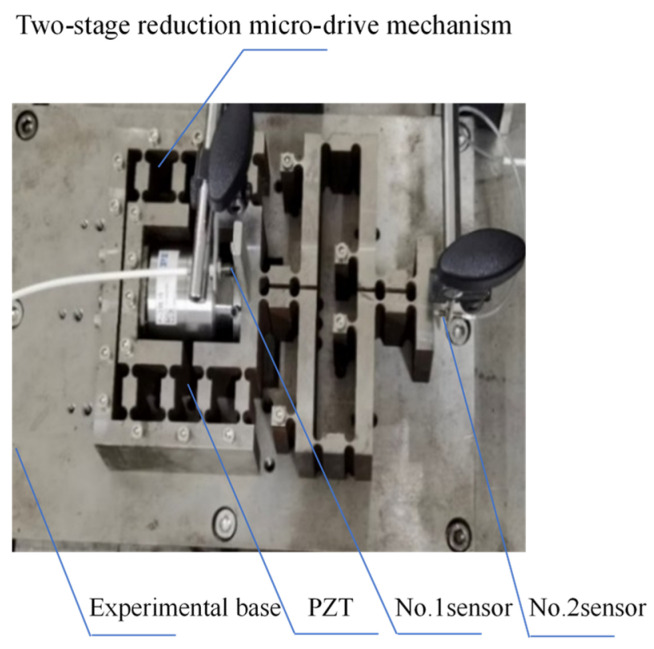
Experiment on motion performance of system.

The motion performance experiment of the two-stage reduction micro-drive mechanism was conducted by using the two-stage reduction micro-drive mechanism, P-235.1s piezoelectric ceramic actuator, eddy current sensor, magnetic meter holder, and experimental base (shown in Figure 11). The two-stage reduction micro-drive mechanism equipped with piezoelectric ceramic actuator was fixed on the experimental base. The input displacement of the mechanism was measured by the No. 1 sensor, and the output displacement of the mechanism was measured by the No. 2 sensor. The experimental data were recorded, and the data obtained are shown in Table 5.

### 4.3. Discussion

#### 4.3.1. Dynamic Performance Analysis

The first six natural frequencies of the secondary reduction micro-drive mechanism were analyzed by finite element and experimental analysis, and the results are shown in Table 4.

Based on the finite element analysis of dynamic performance and experimental results, the following could be observed:

(1) The finite element and experimental analysis results were consistent and the maximum error was 8.14%, indicating that the analysis results were accurate and reliable.
(2) The reduction micro-drive mechanism was driven by P235.1s piezoelectric ceramic actuator, whose maximum frequency is 300 Hz and natural frequency was not resonant with the reduction fretting mechanism, and the two-stage reduction micro-drive mechanism has good dynamic performance.

Therefore, the two-stage reduction micro-drive mechanism had good dynamic performance and met the requirements of design and use.

#### 4.3.2. Motion Performance Analysis

The designed reduction ratio of the two-stage reduction micro-drive mechanism was 25:1. Its reduction performance was analyzed using the finite element method and verified through experimental testing. To validate the accuracy of the reduction ratio, value verification was conducted within the input range of 0–15 μm. The experimental data results were then compared with the finite element analysis results, as presented in Table 5. The data results obtained by the experiment were compared with the finite element analysis results.

**Figure 12:**
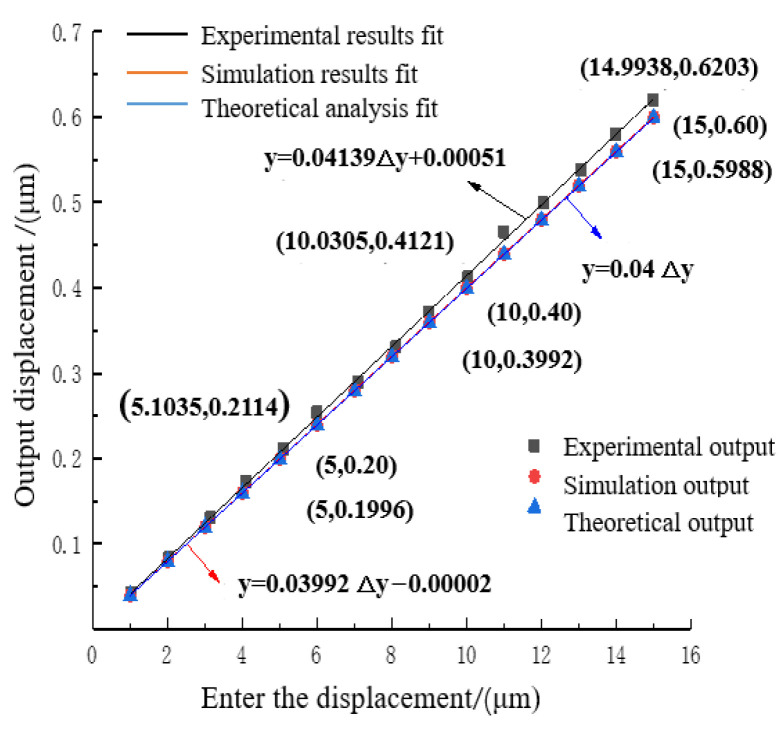
Micro-drive mechanism motion performance linear fitting.

The theoretical output displacement, finite element analysis output displacement, and experimental result output displacement of the two-stage reduced micro-drive mechanism were linearly fitted, as shown in Figure 12.

The linear equation obtained by fitting the theoretical output displacement was

(13) y=0.04Δy

Its linearity was 1.

The linear equation obtained by fitting the output displacement of finite element analysis was

(14) y=0.03992∆y−0.00002 

Its linearity was 1.

The linear equation obtained by fitting the output displacement of the experimental results was

(15) y=0.04139∆y+0.00051

Its linearity was 0.99952.

From the fitted linear Equations (13)–(15), it could be seen that the input displacement Δu and output displacement Δv of the two-stage reduced micro-drive mechanism have high linearity. In order to facilitate practical application, the above fitted linear Equations (13)–(15) combined with the characteristics of the two-stage reduction micro-drive mechanism could be approximated and described by the linear formula of Equation (16).

(16) y=0.0404∆y 

(1) Reduction ratio analysis

From Equations (13)–(15), it was evident that the designed reduction ratio of the two-stage reduction micro-drive mechanism was 25.00:1, the simulated reduction ratio was 25.05:1, and the experimental reduction ratio was 24.16:1. Equation (16) yields a reduction ratio of 24.73:1 for the two-stage reduction micro-drive mechanism.

In comparison to the original institution’s reduction ratio (2.1), the aforementioned reduction ratios increased by 525%, 526.25%, and 504%, respectively, resulting in a comprehensive reduction ratio increased of 518.25%.

The reduction ratio of the micro-drive mechanism was compared with the relevant literature, as presented in Table 6. It could be observed that the designed reduction ratio of the mechanism described in this paper was the smallest in all mechanisms. The reduction ratio of this mechanism was 24.73:1.

(2) Error Analysis

By utilizing the slope error of the fitted linear Equations (13)–(15), the maximum error between the theoretical analysis value and the experimental value was found to be 8.83%. Referring to Table 5, the maximum error was measured to be 0.0258 μm. Similarly, the error between two analysis values was determined to be 9.02%, with a maximum error of 0.0267 μm, according to Table 5.

The maximum motion error of this mechanism was calculated to be 9.02%, with a maximum error value of 0.0267 μm. The motion error of this mechanism was compared to the motion errors of other micro-motion mechanisms discussed in the literature, as shown in Table 7.

It can be seen from Table 7 that the motion error of this mechanisms is the smallest in all mechanisms. Meanwhile, from Equations (13)–(15), the minimum linearity of the two-stage reduction micro-drive mechanism in this paper was calculated as 0.99952, which was relatively high. Therefore, the mechanism had the advantages of a small reduction ratio (24.73:1), high motion accuracy (maximum motion error 0.0267 μm), and high motion linearity (minimum linearity 0.99952).

## 5. Conclusions

This study focused on the design and performance analysis of a two-stage reduction micro-drive mechanism based on particle swarm optimization. The innovation of this paper lies in adopting a two-stage reduction method within a limited space by using the particle swarm optimization algorithm to achieve the maximum reduction ratio. This enables higher-precision motion over a larger range within a smaller motion envelope, thereby providing high-precision motion in small spaces. The main conclusions of this paper are as follows:

(1) The micro-drive mechanism designed in this paper incorporates a hinge structure, which effectively eliminates parasitic motion and non-motion direction forces through the application of a balanced additional force function.
(2) Utilizing particle swarm optimization, the structure of the two-stage reduction micro-drive mechanism was optimized to achieve the minimum reduction ratio. After optimization, finite element and experimental analyses revealed that the maximum simulated stress of the mechanism was 39.681 MPa, significantly lower than the allowable stress of the material.
(3) The first six natural frequencies of the mechanism were found to be non-overlapping with the motion frequency of the micro-driver, guaranteeing the absence of resonance during mechanism operation. Therefore, the strength performance and dynamic performance of the mechanism were excellent and met the design and use requirements.
(4) The two-stage reduction micro-drive mechanism achieved a minimum reduction ratio of 24.73:1, representing a reduction of 518.25% compared to the original design. The motion error of this mechanism was determined to be 9.02%, with a maximum motion error of 0.0267 μm, while the minimum motion linearity reached 0.99952. Consequently, this mechanism possesses characteristics such as a small reduction ratio, high motion accuracy, and superior motion linearity.

Future work related to this paper will be conducted in macro–micro dual-drive systems and systematic error compensation research. The research in this paper has important reference significance for the design and performance optimization of precision micro-drive mechanisms.

## Figures and Tables

**Table 1:** Material parameters of flexure hinge.

Material Name	Young’s Modulus (MPa)	Limit of Yielding (MPa)	Tensile Strength (MPa)	Poisson’s Ratio	Density (g/cm^3^)
60Si2Mn	2.06 × 10^5^	1176	1274	0.26	7.85

**Table 2:** Initial coordinate values for reduction action flexure hinge (unit: mm).

x_1_	x_2_	x_3_	x_4_	x_5_	x_6_
14	40	53	53	29	17

**Table 3:** The first six natural frequencies of the micro-drive mechanism.

Modal Order	1	2	3	4	5
Natural frequency value (Hz)	167.62	196.99	520.44	562.27	705.14

**Table 4:** Dynamic analysis comparison between finite element method and experiment.

Order	Natural Frequency Value of Finite Element Analysis (Hz)	Experimental Natural Frequency Value (Hz)	Relative Error (%)
1	167.62	181.00	7.98%
2	196.99	208.50	5.84%
3	520.44	482.00	7.38%
4	562.27	516.50	8.14%
5	705.14	653.50	7.32%
6	712.98	665.00	6.72%

**Table 5:** Comparison of theoretical results, finite element analysis results, and experimental results.

Enter the Displacement (µm)	Theoretical Output Displacement (µm)	Finite Element Analysis of Output Displacement	Enter the Displacement (µm)
1.0000	0.0400	0.0392	0.0426
2.0000	0.0800	0.0798	0.0835
3.0000	0.1200	0.1198	0.1306
4.0000	0.1600	0.1597	0.1725
5.0000	0.2000	0.1996	0.2114
6.0000	0.2400	0.2395	0.2541
7.0000	0.2800	0.2794	0.2896
8.0000	0.3200	0.3193	0.3305
9.0000	0.3600	0.3593	0.3716
10.0000	0.4000	0.3992	0.4121
11.0000	0.4400	0.4391	0.4658
12.0000	0.4800	0.4791	0.4993
13.0000	0.5200	0.5190	0.5387
14.0000	0.5600	0.5589	0.5797
15.0000	0.6000	0.5988	0.6203

**Table 6:** Comparing reduction ratio with that of other precision mechanisms.

Reference	Author	Year	Reduction Ratio
[30]	Sakuma S. et al.	2013	7.50:1
[19]	Yang M. et al.	2022	2.00:1
[39]	Chen Y. et al.	2022	7.30:1
This Paper	Yang M. et al.	2023	24.73:1

**Table 7:** Comparing motion error with that of other precision mechanisms.

Reference	Author	Year	Error (µm)
[19]	Yang M. et al.	2022	0.3700
[33]	Chen F. et al.	2022	0.1000
[40]	Wang G. et al.	2020	4.6000
This paper	Yang M. et al.	2023	0.0267

## Data Availability

The data presented in this study are available on request from the corresponding author.
